# Dietary iodine attenuates allergic rhinitis by inducing ferroptosis in activated B cells

**DOI:** 10.1038/s41598-023-32552-1

**Published:** 2023-04-03

**Authors:** Yutaka Nakamura, Yozen Fuse, Seiga Komiyama, Takahiro Nagatake, Jun Kunisawa, Koji Hase

**Affiliations:** 1 Division of Biochemistry, Faculty of Pharmaceutical Science, Keio University, Tokyo, 105-8512 Japan; 2 Research Committee on Iodine-Related Health Problems, Foundation for Growth Science, Tokyo, 113-0033 Japan; 3 Laboratory of Functional Anatomy, Department of Life Sciences, School of Agriculture, Meiji University, Kanagawa, 214-8571 Japan; 4 Laboratory of Vaccine Materials, Center for Vaccine and Adjuvant Research and Laboratory of Gut Environmental System, Collaborative Research Center for Health and Medicine, National Institutes of Biomedical Innovation, Health and Nutrition, Osaka, 567-0085 Japan; 5 The Institute of Fermentation Sciences (IFeS), Faculty of Food and Agricultural Sciences, Fukushima University, Kanayagawa, Fukushima, 960-1296 Japan; 6 International Research and Development Centre for Mucosal Vaccines, The Institute of Medical Science, The University of Tokyo (IMSUT), Tokyo, 108-8639 Japan

**Keywords:** Immunology, Adaptive immunity, Mucosal immunology

## Abstract

Iodine-containing formulations have been widely used to treat iodine deficiency and as antiseptics. Lecithin-bound iodine (LBI) has been approved to treat allergic diseases in Japan; however, its underlying mechanism remains unknown. In this study, we show that LBI ameliorated disease symptoms in an ovalbumin (OVA)-induced allergic rhinitis mouse model. LBI suppressed OVA-specific IgE production by attenuating germinal center (GC) reaction in the draining lymph nodes. The antiallergic effect of LBI is most likely attributed to increased serum iodine levels but not thyroid hormone levels. In vitro treatment of activated B cells with potassium iodide induced ferroptosis by increasing intracellular reactive oxygen species (ROS) and ferrous iron in a concentration-dependent manner. Accordingly, LBI diets increased ROS levels in GC B cells of the draining lymph nodes. This study suggests that iodine directly promotes ferroptosis in activated B cells and attenuates GC reactions, leading to the alleviation of allergic symptoms.

## Introduction

Trace element iodine is an essential component of the thyroid hormones triiodothyronine (T3) and thyroxine (T4), which promote basal metabolism, lipid metabolism, and protein synthesis. In addition, thyroid hormones influence immune cell functions, such as dendritic cell maturation, macrophage polarization, apoptosis, and lymphocyte proliferation^1–5^. Iodine deficiency results in the loss of thyroid hormone production, thereby inducing hypothyroidism. Interestingly, a pilot study showed that approximately 20% of patients with allergic rhinitis had hypothyroidism^6^, suggesting that dietary iodine deficiency is a potential predisposing factor for allergic rhinitis.

Lecithin-bound iodine (LBI), synthesized from purified soybean lecithin and iodine, has been approved for the treatment of childhood asthma and thyroid diseases in Japan. LBI-derived iodine is absorbed in the upper gastrointestinal tract as iodide and increases serum iodide levels within 2 h in humans^7^. Administration of LBI to house dust mite-stimulated human peripheral blood mononuclear cells (PBMCs) increases interferon-γ (IFN-γ) production, resulting in decreased interleukin (IL)-4 and IgE levels^8,9^. Similarly, treatment with sodium iodide in human leukocytes induces the release of several cytokines, including IFN-γ^10^. These reports suggest that LBI may regulate the balance between T helper 1 (Th1) and T helper 2 (Th2) cells in allergic diseases. However, the underlying mechanisms of LBI-mediated antiallergic effects remain unknown.

Ferroptosis, a type of non-apoptotic programmed cell death, is characterized by iron-dependent lipid peroxidation caused by lipoxygenases (e.g., Alox5 and Alox15) and the Fenton reaction^11–13^. Nutrients such as trace elements, vitamin E, and coenzyme Q10, can positively or negatively regulate ferroptosis induction^14^. For example, ferrous iron reduces hydroxyl peroxide and produces hydroxyl radicals through the Fenton reaction to promote ferroptosis^15^. In murine hepatocytes, excess administration of iron promotes ferroptosis^16^. In addition, selenium serves as a cofactor of glutathione peroxidase 4 (Gpx4) that inhibits lipid peroxidation, resulting in the attenuation of ferroptosis^17^. Oral administration of selenium increases GPX4 expression and inhibits cell death in T follicular helper (Tfh) cells by alleviating the generation of reactive oxygen species (ROS)^18^. However, whether the antiallergic effect of iodine supplementation is mediated by the regulation of ferroptosis remains unknown.

This study aimed to investigate the mechanisms by which LBI exhibits its antiallergic effects. Our results indicated that LBI-containing diets alleviate ovalbumin (OVA)-induced allergic rhinitis, accompanied by reduced OVA-specific IgE in the serum. Additionally, we revealed that in LBI-fed mice, the serum levels of iodine, but not thyroid hormones, increased. Exposure of activated B cells to potassium iodide (KI) promoted cell death with increased ROS, ferrous iron, and membrane permeability in a dose-dependent manner. In addition, cell death in KI-exposed activated B cells was inhibited by ferrostatin-1 (Fer-1), a popular inhibitor of ferroptosis^15^. We also observed increased ROS production in B cells in the draining lymph nodes (dLNs) of LBI-fed mice. Therefore, this study suggests that increased dietary iodine suppresses the activation of B cells by promoting ferroptosis, resulting in the inhibition of allergen-specific IgE and the subsequent suppression of allergic symptoms.

## Results

### LBI-rich diet alleviates the nasal symptom of allergic rhinitis

**Figure 1:**
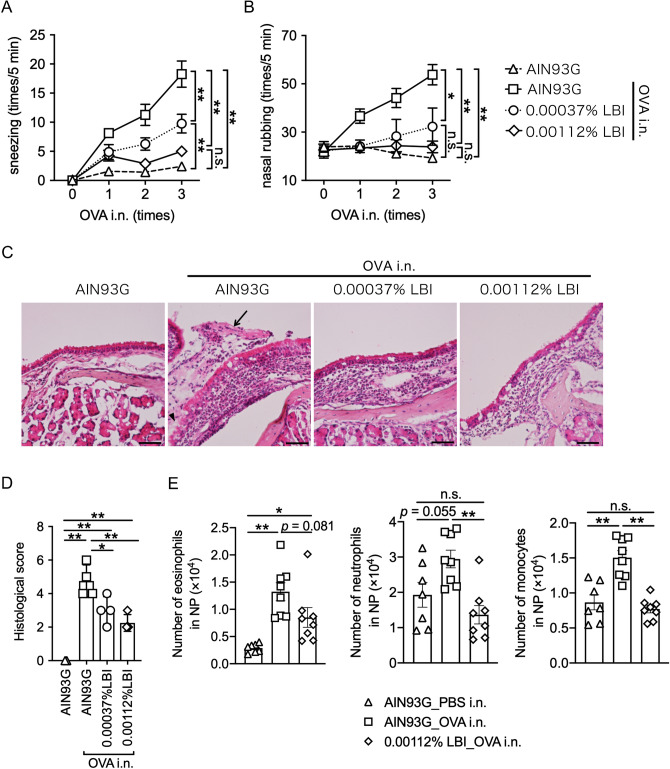
LBI supplementation attenuates OVA-induced allergic symptoms and histological damage in mice. (**A**–**E**) Allergic rhinitis was induced by OVA exposure in pre-sensitized male C57BL/6J mice. Sneezing (**A**) and nasal rubbing (**B**) were counted for 5 min immediately after the administration of intranasal PBS or OVA exposure. The data were pooled from two different experiments and are shown as the means ± SEM (*n* = 7, 8, 8, 8). (**C**) Representative histological images of nasal mucosa stained with hematoxylin and eosin (**C**). (**D**) Assessment of histological damage using histological damage scoring (**D**). Data are presented as the means ± SD (*n* = 3, 4, 4, 4). Scale bar, 50 μm. (**E**) The number of Siglec-F^+^CD11b^+^CD45^+^eosinophils, Siglec-F^-^CD11b^+^CD45^+^Ly6G^+^Ly6C^-^neutrophils, and Siglec-F^-^CD11b^+^CD45^+^Ly6G^-^Ly6C^+^monocytes in the nasal passage. The data were pooled from two different experiments and shown as the means ± SEM (*n* = 7, 8, 8). Two-way analysis of variance ANOVA test with Tukey’s multiple comparisons test (**A**, **B**). One-way ANOVA test with Tukey’s multiple comparisons test (**D**–**E**). n.s.: not significant; *p < 0.05, **p < 0.01. OVA, ovalbumin; PBS, phosphate-buffered saline; NP, nasal passage.

To determine the effects of LBI on allergic diseases in mice, we supplemented LBI to AIN93G-based purified diets at a clinically approved dose (0.00037%) or a three-fold higher dose (0.00112%). AIN93G- or LBI-fed C57BL/6J mice received intraperitoneal sensitization with OVA/alum (ovalbumin/aluminum) three times, followed by an intranasal challenge (three times) with the same antigen to induce allergic rhinitis (Figure S1A). The symptoms of allergic rhinitis in AIN93G-fed control mice gradually increased, with increased sneezing and nose rubbing counts during repeated intranasal challenges. In contrast, these nasal symptoms were alleviated in a dose-dependent manner in both LBI groups (Fig. 1A,B). Histological examination also confirmed that LBI diets attenuated severe epithelial shedding, cell infiltration, and exudation in the respiratory mucosa compared to the AIN93G group (Fig. 1C,D). Furthermore, flow cytometry demonstrated that the 0.00112% LBI diet resulted in a reduced number of eosinophils, neutrophils, monocytes (Figs. 1E and S1B), and T cell subsets (Figure S2A) in the nasal passage and Tfh cells in dLNs (Figures S2B). In addition, although a previous study reported increased levels of IFN-γ in in vitro PBMCs cultured with LBI^8,9^, in our study, serum IFN-γ levels were comparable among all groups (Figure S1C). These data indicate that LBI supplementation alleviated OVA-induced allergic rhinitis.

### LBI increases serum iodine, but not thyroid hormones, in mice

**Figure 2:**
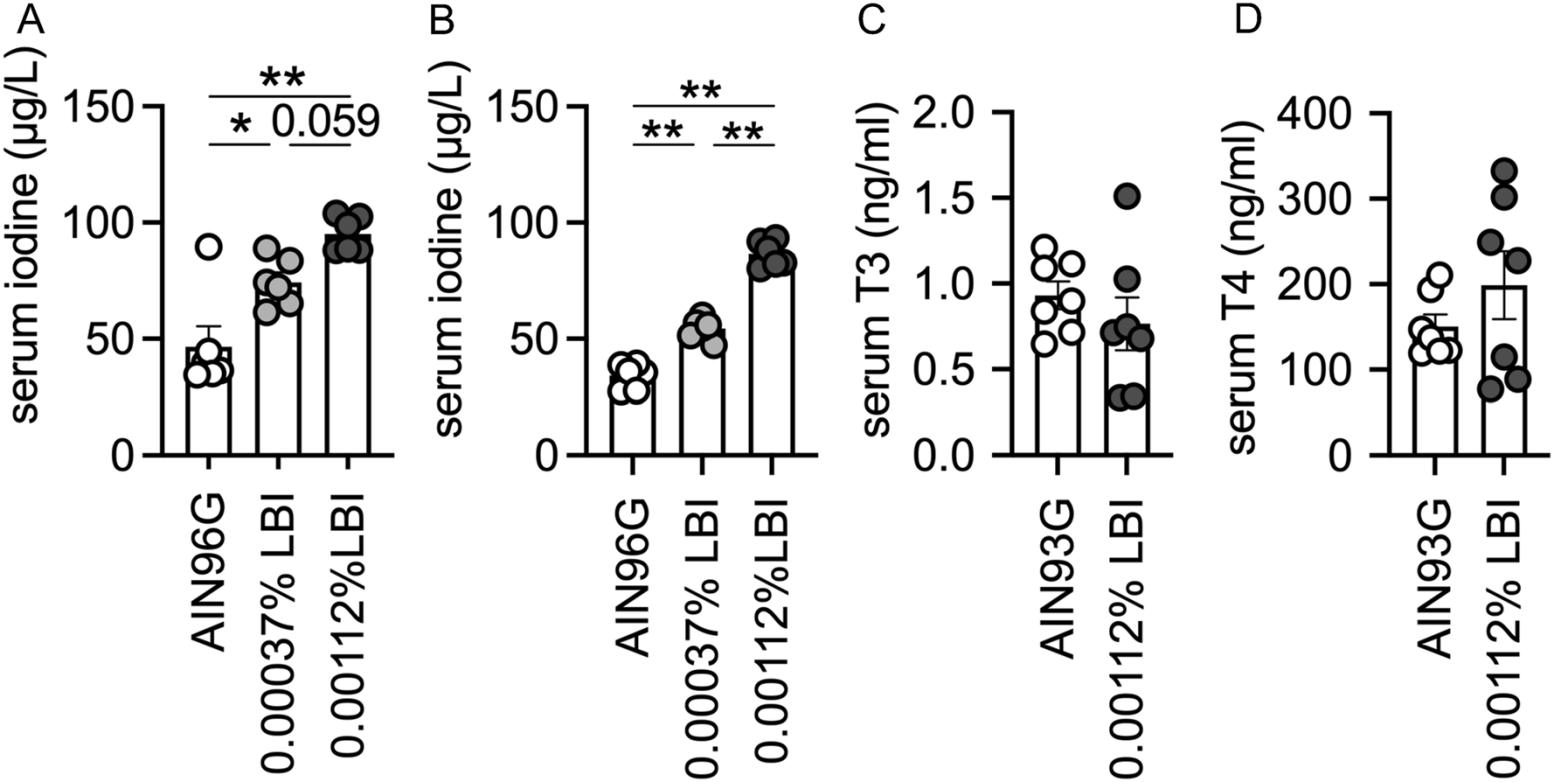
LBI increases serum iodine levels in mice. (**A**, **B**) The concentration of serum iodine was measured on days 7 (**A**) or 21 (**B**) of LBI feeding. The data were pooled from two independent experiments and shown as the means ± SEM (*n* = 6 for each group). (**C**, **D**) The concentrations of triiodothyronine (T3) (**C**) and thyroxine (T4) (**D**) in serum were measured on day 7 of LBI feeding. Data are pooled from two independent experiments and shown as the means ± SEM (*n* = 7 for each group). One-way ANOVA test with Tukey’s multiple comparisons test (**A**, **B**). Student’s *t*-test (**C**). Welch’s *t*-test (**D**). n.s., not significant; *p < 0.05, **p < 0.01.

As iodine is an essential component of thyroid hormones, we investigated whether LBI diets affect the serum levels of total iodine or iodide and thyroid hormones. We observed that after one week of feeding, the serum levels of total iodine were increased in a dose-dependent manner in the LBI groups (Fig. 2A), and a similar tendency was sustained after three weeks of feeding (Fig. 2B). Thus, LBI feeding provided a stable supply of high doses of circulating iodine. In contrast, there were no significant differences in serum T3 and T4 levels between LBI- and AIN93G-fed mice (Fig. 2C,D). Similarly, there were no significant differences in the hepatic expression of the thyroid hormone-responsive genes *Thrsp* and *Me1*^19^ between mice fed AIN93G or LBI diets for three weeks (Figure S3A and B). These results suggest that LBI most likely exerts an antiallergic effect by increasing the serum level of iodine rather than thyroid hormones.

### LBI supplementation reduces the levels of OVA-specific IgE in mice

**Figure 3:**
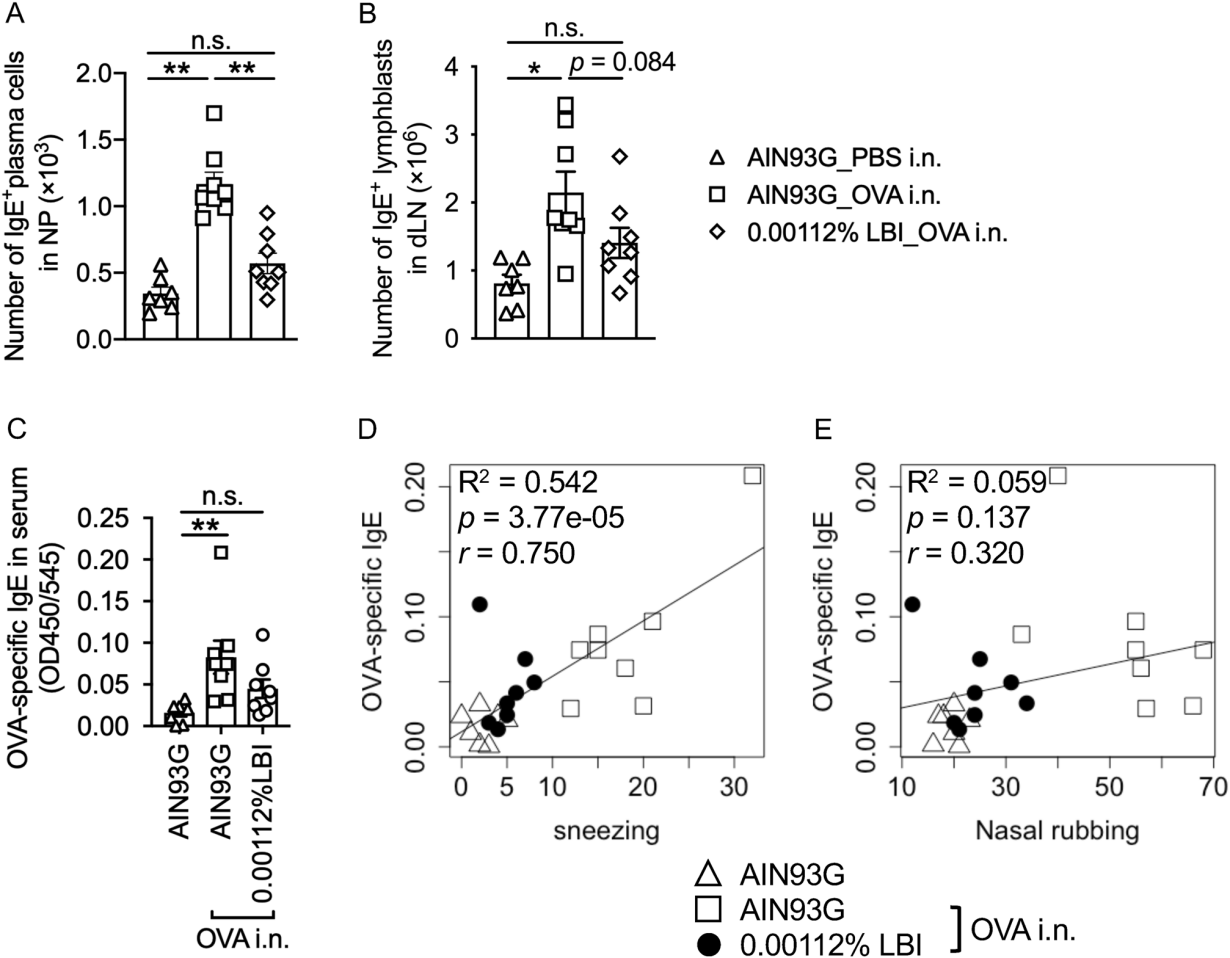
LBI supplementation reduced the levels of OVA-specific IgE in OVA-induced allergic rhinitis mice. (**A**, **B**) The number of IgE^+^B220^-^CD45^+^ IgE-producing plasma cells in the nasal passage (**A**) and IgE^+^B220^+^CD45^+^ IgE-class-switched lymphoblasts in the draining lymph nodes (**B**). (**C**) Enzyme-linked immunosorbent assay of OVA-specific IgE. The correlation between IgE levels and nasal symptoms (**D**: sneezing, **E**: nasal rubbing) was calculated using Pearson’s test. The data were pooled from two different experiments and are shown as the mean ± SEM (*n* = 7, 8, 8). One-way ANOVA test with Tukey’s multiple comparisons test (**A**, **B**). Kruskal–Wallis test (**C**). n.s.: not significant; *p < 0.05, **p < 0.01. NP, nasal passage; dLN, draining lymph nodes.

To investigate the underlying mechanism of the LBI-mediated antiallergic effects, we compared the levels of IgE in mice. The results revealed that the number of IgE^+^ plasma cells was decreased in the nasal passage of LBI-fed mice compared to the control and AIN93G groups (Fig. 3A). A similar tendency was observed in IgE^+^B220^+^ lymphoblasts in the draining lymph nodes of LBI-fed mice (Fig. 3B). These observations imply that LBI may suppress allergic rhinitis by reducing IgE production. Indeed, the levels of OVA-specific IgE were significantly increased in AIN93G-fed diseased mice, but not in 0.00112% LBI-fed diseased mice, compared to the control mice (Fig. 3C). In addition, the OVA-specific IgE levels correlated well with the number of sneezes (*r* = 0.750; Fig. 3D). In contrast, the number of nasal rubbings had a lower correlation coefficient (*r* = 0.320) with OVA-specific IgE levels (Fig. 3E). These results suggest that LBI may suppress the induction of OVA-specific IgE to alleviate nasal symptoms, especially sneezing, in allergic rhinitis.

### Iodide supplementation decreases the viability of activated B cells

**Figure 4:**
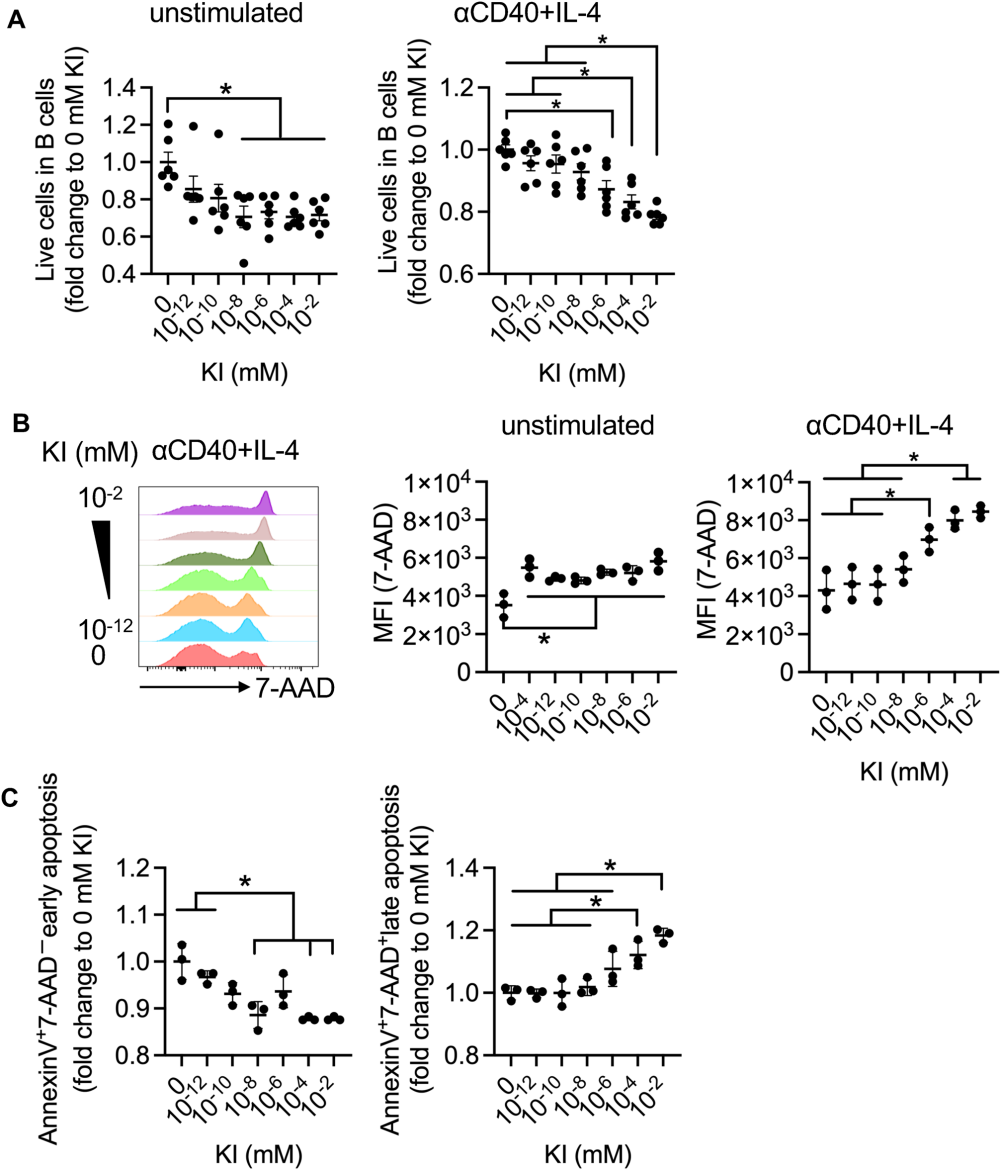
A high dose of iodide promotes activated B cell death via non-apoptotic cell death. Splenic B cells collected from C57BL/6 J mice were cultured with anti-CD40 antibody, IL-4, and serially diluted KI for three days. (**A**) The relative survival rate of B cells (compared to that of untreated cells). The data were pooled from two independent experiments and are shown as mean ± SEM (*n* = 6 for each point). (**B**) Median fluorescence intensity of 7-AAD. Representative data from two independent experiments are shown as the mean ± SD (*n* = 3 for each point). (**C**) Annexin V^+^7-AAD^-^ early apoptotic B cells (left) and annexin V^+^7-AAD^+^ late apoptotic B cells (right). Representative data from two independent experiments are shown as the mean ± SD (*n* = 3 for each point). One-way ANOVA with Tukey’s multiple comparison test. n.s.: not significant, *p < 0.05, **p < 0.01. IL, interleukim; KI, potassium iodide.

Because LBI feeding attenuated IgE levels in the allergic rhinitis model, we further analyzed the direct effect of iodide on the transition of B cells to IgE-producing plasma cells. However, KI supplementation did not affect the frequency of IgE^+^ B cells among B220^+^ total B cells (Figure S4A and B). In contrast, the survival rate of activated B cells upon stimulation with an anti-CD40 monoclonal antibody (mAb) decreased by treatment with KI in a dose-dependent manner (Fig. 4A). Likewise, activated B cells with higher concentrations of KI showed an enhanced median fluorescence intensity (MFI) of 7-AAD, indicative of increased plasma membrane permeability. Notably, KI supplementation only slightly increased the 7-AAD signal in non-activated (unstimulated) B cells (Fig. 4B). These data demonstrate that iodide supplementation may directly induce cell death in activated B cells without affecting IgE class-switching.

### Iodide supplementation induces ferroptosis in activated B cells

**Figure 5:**
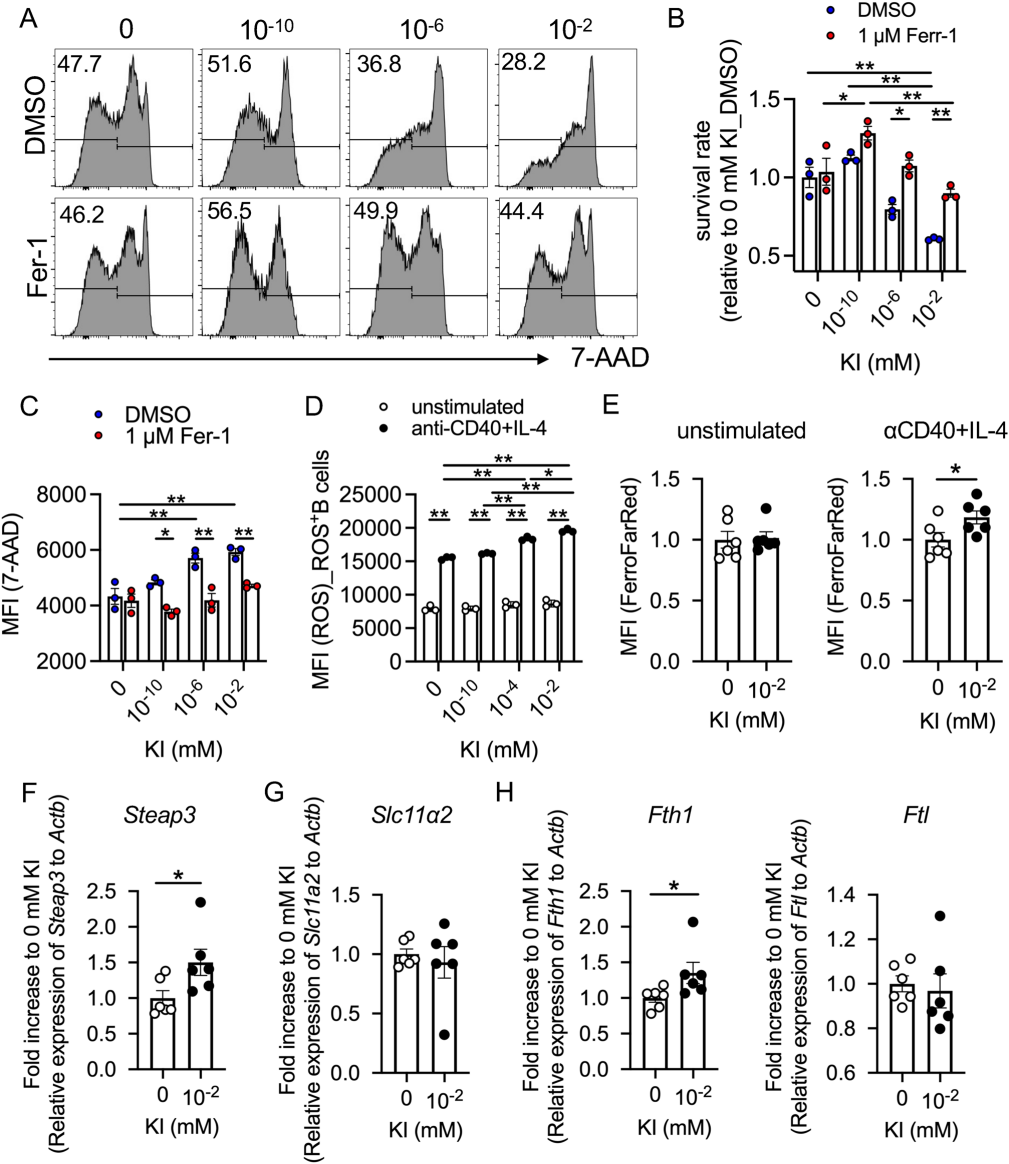
KI promotes ferroptosis of activated B cells. Splenic B cells collected from C57BL/6 J mice were cultured with anti-CD40 antibody, IL-4, and serially diluted KI for three days. (**A**) Histogram of 7-ADD expression in B cells stimulated with DMSO or Fer-1. (**B**,**C**) Survival rate (**B**) and MFI of 7-AAD (**C**) in stimulated B cells with DMSO or Fer-1. Representative data from two independent experiments are shown as the mean ± SD (*n* = 3 for each point). (**D**) MFI of ROS in the ROS^+^B220^+^CD45^+^B cells. Representative data from two independent experiments are shown as the mean ± SD (*n* = 3 for each point). (**E**) Intracellular ferrous iron measurements in FerroFarRed^+^ cells cultured with or without anti-CD40 and IL-4 for 3 days. (**F**–**I**) Quantitative polymerase chain reaction analysis of the relative expression levels of *Steap3* (**F**), *Slc11a2* (**G**), *Fth1*, and *Ftl1* (**H**) in anti-CD40-stimulated B cells. Data were pooled from two independent experiments and are shown as mean ± SEM (*n* = 6 for each point). Welch’s *t*-test. n.s.: not significant, *p < 0.05, **p < 0.01. IL, interleukin; KI, potassium iodide; MFI, mean fluorescence intensity; ROS, reactive oxygen species.

Cell death is categorized into several types, including apoptosis, necrosis, and ferroptosis, which are mediated by different mechanisms. To characterize the iodide-dependent cell death, we initially detected cell surface phosphatidylserine, a marker of apoptosis, by Annexin V. The results revealed that KI supplementation to activated B cells increased the Annexin V^+^7-AAD^+^ late apoptotic cell population, but not the Annexin V^+^7-AAD^-^ early apoptotic cell population (Fig. 4C and Figure S5). The increase in the late apoptotic population is suggestive of necrosis or ferroptosis, suggesting that KI-induced B cell death may be mediated by a non-apoptotic pathway. To investigate this possibility, we treated B cells with KI and an inhibitor of necrosis (necrosulfonamide), pyroptosis (z-YVAD-fmk), or ferroptosis (Ferrostatin-1: Fer-1). Consequently, only Fer-1 rescued KI-induced cell death and maintained membrane integrity (Figs. 5A–C and S6A,B), indicating that iodide promoted ferroptosis in activated B cells.

We further observed that stimulation of B cells with anti-CD40 mAb promoted the accumulation of ROS, which was further exacerbated by treatment with KI (Fig. 5D). In addition, ferrous iron (Fe^2+^) is critical for lipid peroxidation because it facilitates the Fenton reaction that generates hydroxyl radicals from hydrogen peroxide. We observed that intracellular ferrous iron increased in activated, but not intact, B cells upon treatment with a high concentration (10^–2^ mM) of KI (Fig. 5E). Accordingly, treatment with KI upregulated the gene expression level of the metalloreductase Steap3 (Fig. 5F), which converts ferric iron to ferrous iron in the endosome, in activated B cells. There was no significant change in the expression levels of the ferrous iron transporter Dmt1 encoded by *Slc11a2*^11^ (Fig. 5G). We also analyzed the heavy chain (*Fth1*) and light chain (*Ftl1*) of ferritin and found that treatment with KI upregulated *Fth1* expression in activated B cells (Fig. 5H). Notably, KI did not affect the expression levels of genes encoding lipoxygenases (*Alox5* and *Alox15*) or glutathione peroxidase (*Gpx4*) (Figure S6C). Collectively, these results indicate that iodide may increase intracellular ferrous iron, leading to the accumulation of ROS and increased ferroptosis.

### LBI diets increase intracellular ROS in B cells

**Figure 6:**
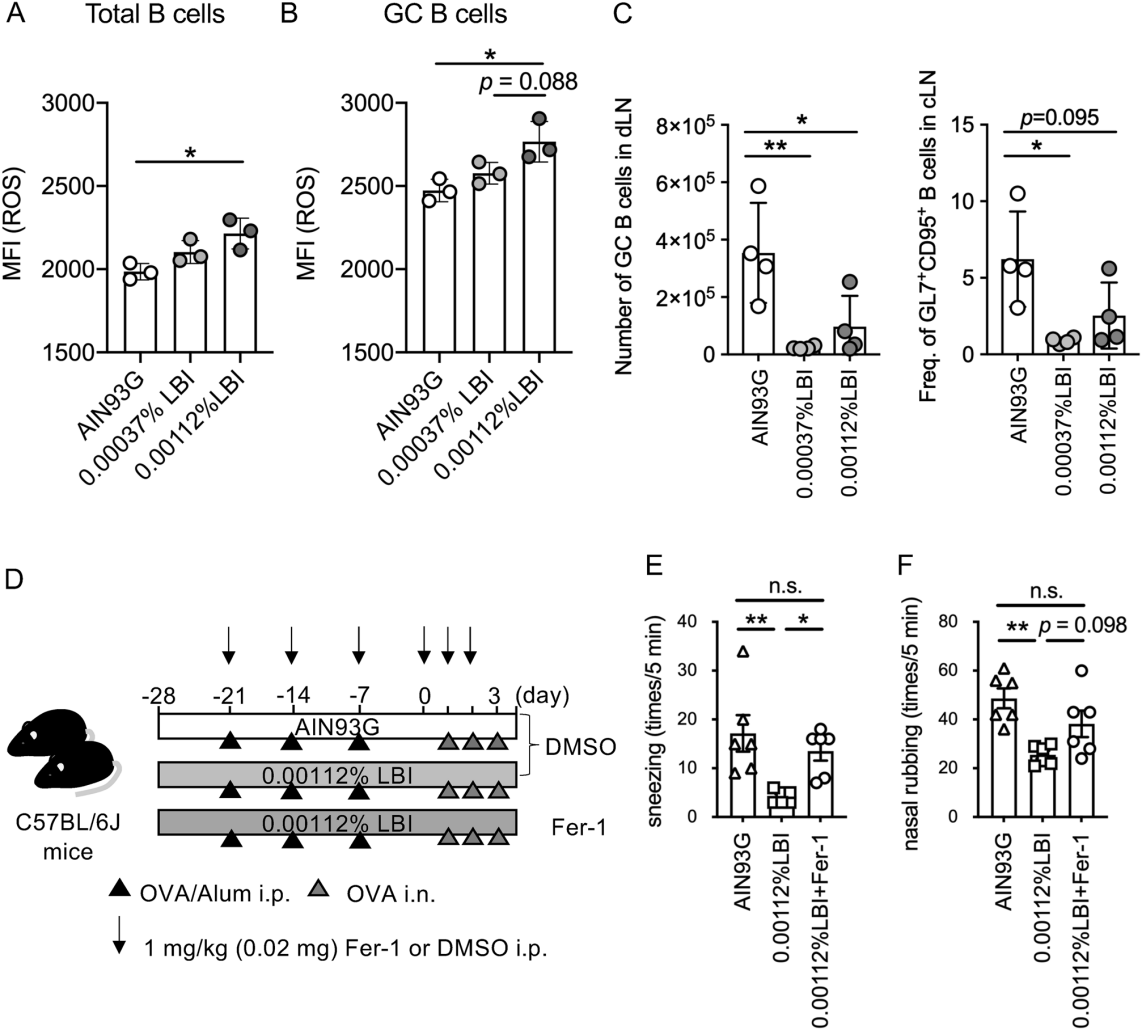
LBI increases the levels of reactive oxygen species ROS in the B cells of draining lymph nodes at a steady state. (**A**, **B**) Male C57BL/6 J mice were fed AIN93G, 0.00037% LBI, or 0.00112% LBI diets for one week. MFI of ROS in the total (**A**) or GL7^+^CD95^+^CD45^+^B220^+^CD3ε^-^germinal center B cells (**B**) of draining lymph nodes. The representative data of two independent experiments are shown as the means ± SD (*n* = 3 for each point). (**C**) Male C57BL/6 J mice were fed AIN93G, 0.0037% LBI, and 0.00112% LBI diets for 3 weeks. The number and frequency of GL7^+^CD95^+^CD45^+^B220^+^CD3ε^-^germinal center B cells in draining lymph nodes. (**D**) A scheme of the experimental protocol of allergic arthritis model. Fer-1 or DMSO was intraperitoneally injected in LBI-fed mice. (**E**, **F**) Frequency of sneezing (**E**) and nasal rubbing (**F**) were evaluated for 5 min immediately after the administration of intranasal PBS or OVA exposure at day 3. The data were pooled from two different experiments and are shown as the means ± SEM (n = 6 for each group). One-way ANOVA test with Tukey’s multiple comparisons test. n.s.: not significant, *p < 0.05, **p < 0.01. MFI, mean fluorescence intensity; ROS, reactive oxygen species.

To confirm that LBI induces cell death in the draining lymph nodes, we analyzed the intracellular ROS levels in B cells. Similar to the in vitro B cell culture system, one-week feeding with LBI increased intracellular ROS levels in B cells (Fig. 6A). In particular, germinal center (GC) B cells, composed of activated B cells, abundantly produced intracellular ROS, which was further elevated by LBI feeding (Fig. 6B). Additionally, three-week feeding of LBI prominently decreased the number and frequency of GC B cells in the draining lymph nodes (Fig. 6C). These data indicate that the oral administration of iodine most likely affects the survival of activated B cells by inducing ferroptosis. Similar to the GC B cells, Tfh cells were also reduced by LBI feeding (Figure S7).

Finally, to confirm the significance of ferroptosis induction in LBI-dependent anti-allergic effect, we intraperitoneally administrated Fer-1 in LBI diet-fed mice (Fig. 6D). Fer-1 treatment canceled the effect of LBI in sneezing and tended to aggravate nasal rubbing (Fig. 6E,F). Thus, ferroptosis induced by LBI may alleviate the nasal symptoms of allergic rhinitis.

## Discussion

We here revealed that LBI alleviates disease symptoms in murine allergic rhinitis by attenuating immune cell infiltration into the nasal mucosa and decreasing the serum allergen-specific IgE levels. To the best of our knowledge, this is the first report showing that iodine alleviates allergic responses in the upper airway, raising the possibility that LBI may provide therapeutic benefits to patients with allergic rhinitis.

In this study, LBI diets increased the serum levels of iodide, but not thyroid hormones. These results are consistent with those of a human study demonstrating that serum levels of thyroid hormones were intact during four weeks of LBI intake^20^. Thyroid hormones are rigorously regulated by a feedback mechanism in the thyrotropin-releasing hormone–thyroid stimulating hormone axis. In addition, in healthy individuals, only 10% of dietary iodine accumulates in the thyroid gland whereas up to 80% of iodine is transferred to the thyroid gland during iodine deficiency^21,22^. Therefore, additional supplementation with iodide (approximately 60 μg/kg) may not influence thyroid hormone levels in mice. These results suggest the production of thyroid hormones is dispensable for the antiallergic effect of LBI.

In addition, our results indicated that treatment with KI promoted ferroptosis and disrupted plasma membrane integrity in activated B cells. Furthermore, LBI intake suppressed the OVA-specific IgE response by reducing GC B cells. Similarly, an early study has shown that B cell-specific deficiency in GPX4 induces ferroptosis in B1 and marginal zone B cells, attenuating IgM responses to *Streptococcus pneumonia*^23^. These data indicate that ferroptosis of activated B cells affects the humoral immune response. Moreover, we observed that treatment with KI upregulated the expression of ferroptosis-related genes*, Steap3* and *Fth1,* accompanied by increased levels of intracellular ferrous iron in activated B cells. Steap3 reduces ferric iron to ferrous iron to promote the Fenton reaction, which is a critical pathway in ferroptosis. *Steap3* gene expression is regulated by several stimuli. For example, *p53*, a gene that responds to cellular stress and DNA injury, directly upregulates *Steap3*^24^. Alternatively, oxidative stress also induces *Steap3*^25^. However, the pathway contributing to iodide-dependent Steap3 induction remains unknown. *Fth1* expression levels are often correlated with *Steap3* expression^26,27^. These findings suggest that our observation of the upregulation of *Fth1* expression by KI may be attributed to increased *Steap3* expression levels. Notably, iodide chemically converts ferric iron to ferrous iron and generates hydroxyl radicals from hydrogen peroxide^28^. Collectively, these results indicate that iodide may facilitate the Fenton reaction by upregulating *Steap3* and directly interacting with ferric iron in activated B cells (Figure S6).

Ferroptosis diminishes the germinal center reaction by reducing not only activated B cells but also Tfh cells that interact with B cells to induce class-switch recombination and affinity maturation. Moreover, the inhibition of ferroptosis increases Tfh cells and enhances humoral immune responses during vaccination^18^. Activated lymphocytes abundantly produce ROS during the proliferation (Fig. 5D)^29,30^. Considering that leukocyte activation by PMA-Ionomycin induced an iodide shuttle-associated receptor PENDRIN expression^10^, we speculate that iodide may also induce ferroptosis in activated Tfh cells as well as GC B cells through the similar mechanism. However, further studies are required to verify this.

Previous ex vivo studies have shown that LBI may also regulate the Th2 response by activating the Th1 response, as evidenced by the increased IFN-γ levels in PBMCs derived from patients with asthma^8,9^. However, in the present study on murine allergic rhinitis in mice, there was no significant difference in the serum levels of IFN-γ between the LBI and control groups. This apparent discrepancy may result from different experimental conditions. In the previous study, the PBMCs from mite-sensitive patients were re-stimulated by mite antigens, whereas we measured serum IFN-γ levels in OVA-induced allergic mice. Alternative, but the not mutually exclusive, interpretation is that the mite antigens are able to stimulate innate immunity via TLR4^31^, which may potentiate Th1 response. We also cannot completely exclude the possibility that LBI might regulate the local production of IFN-γ in the nasal tissue or lymph nodes. In addition, iodine intake may also affect the functions of nasal epithelial cells because LBI protects the retinal pigment epithelial cell line ARPE-19 against hypoxic stress by maintaining tight junction integrity^32^. Therefore, iodine intake may protect against allergic diseases by promoting multiple host defense mechanisms. Meanwhile, it remains unknown whether LBI affects effector functions of other immune cell subsets, such as mast cells that play a vital role in type I allergic reaction. It is interesting to analyze the effect of LBI on the degranulation and histamine release in mast cells in the future.

One of the leading causes of hypothyroidism in the modern world is Hashimoto’s thyroiditis, an autoimmune disease that generates antithyroid autoantibodies. Hypothyroidism is a potential risk factor for allergic rhinitis^6^. In contrast, patients with Graves’ disease, an autoimmune disease that generates anti-TSH autoantibodies and causes hyperthyroidism, are highly susceptible to seasonal allergic diseases (42.9% in patients with Graves’ disease; 32.6% in healthy controls)^33^. These reports imply that abnormalities in thyroid function may be dispensable for the development of allergic rhinitis. In addition, accumulating evidence suggests that iodine may exert extrathyroid effects^34,35^. Therefore, dietary iodine may help prevent the development of allergic diseases by regulating immune responses. The ferroptosis-inducing activity of LBI may raise the possibility that LBI attenuate host defense functions against infectious agents. Nevertheless, no reports have claimed such an adverse effect of LBI so far. Because excess iodine is readily excreted in the urine^21^, the adverse effect of LBI may be limited in vivo*.*

In conclusion, we propose that iodine alleviates disease symptoms in an ovalbumin allergic rhinitis mouse model by suppressing antigen-specific IgE response by inducing ferroptosis in activated B cells. Excessive activation of B cells is observed in not only allergic diseases but also certain autoimmune disorders. Therefore, it is possible to speculate that iodine supplementation may also regulate autoimmunity, although further investigations will be necessary to verify this possibility.

## Materials and methods

### LBI diets and animals

Considering the clinical dosage of LBI in humans (10–20 μg iodine/kg/day), we prepared AIN93G diets containing 0.00038% or 0.00112% LBI, which is equivalent to 20 or 60 μg iodine/kg/day, respectively (Oriental Yeast Co. LTD, Tokyo, Japan). The iodine concentrations in LBI, 20 μg/kg (0.00038%), and 60 μg/kg (0.00112%), were determined by the general food intake (2 g/day) of a 25 g adult mouse and content rate of iodine in lecithin-bound iodine (6.7%).

C57BL/6 J mice were purchased from CLEA Japan (Tokyo, Japan). The mice were maintained under conventional conditions or specific-pathogen-free conditions and fed AIN93G, 0.00038% LBI/AIN93G, or 0.00112% LBI/AIN93G from 4 to 6 weeks old. All animal experiments were approved by the Animal Research Committee of Keio University.

### OVA-induced allergic rhinitis model

The LBI diets were provided to the mice one week before immunization or three days before the intranasal OVA challenge. OVA-induced rhinitis was induced according to a previously established protocol^36,37^. Briefly, OVA (25 μg) (Sigma-Aldrich, St. Louis. MO, USA) with 1 mg alum (Sigma-Aldrich) was intraperitoneally injected into the mice once a week three times. One week after the last immunization, sneezing and nasal rubbing were assessed for 5 min after intranasal administration of 0.5 mg OVA (25 mg/ml OVA in 20 μl PBS). OVA was intranasally administered daily from days 1 to 3. Phosphate-buffered saline (PBS) was administered intranasally to the healthy control group.

In a separate experiment, 1 mg/kg Fer-1 (0.2 mg/ml in 2% DMSO; 100 μl/mouse) or equal amount of vehicle was intraperitoneally injected with OVA/Alum after the evaluation of sneezing and nasal rubbing.

### Histology

Mouse heads were fixed with 4% paraformaldehyde (PFA) overnight and decalcified with Osteomoll according to the manufacturer’s instructions (MilliporeSigma, Burlington, MA, USA). The decalcified tissues were placed in 30% sucrose overnight and embedded in the O.C.T. compound. The 5 μm specimens were stained with hematoxylin and eosin. Histological scores were calculated based on the status of epithelial cells: (0) normal, (1) loss of microvilli, (2) part shedding, (3) complete shedding, and cell infiltration; (0) normal, (1) mild, (2) severe, and (3) exudate to the lumen.

### Enzyme-linked immunosorbent assay (ELISA) and enzyme immunoassay (EIA)

Mouse serum was collected within 24 h of the last intranasal OVA administration in the experimental rhinitis model. Total IgE and OVA-specific IgE were detected using ELISA MAX™ Standard Set Mouse IgE (BioLegend, San Diego, CA, USA) and LEGEND MAX™ Mouse OVA-specific IgE ELISA Kit (BioLegend) following the manufacturer’s instructions. A triiodothyronine ELISA kit (LifeSpan BioSciences, Seattle, WA, USA) and thyroxine (T4) EIA kit (Arbor Assays, Ann Arbor, MI, USA) were used to detect serum triiodothyronine and thyroxin, respectively, according to the manufacturer’s instructions.

### Measurement of serum iodine levels

Iodine measurements were performed as previously described^38^. Serum samples were prepared by diluting 100 µL of serum with 5 mL of a diluent containing 0.5% TMAH, tellurium (Te), and Indium (In) (SCP Science, Quebec, Canada). Iodine standard 1000 μg/mL sodium iodide (AS-I9-2Y, Lot No. 4-25I-2Y; SPEX CertiPrep, Metuchen, NJ, USA) was used as the reference material for iodine. To avoid signal drift, Te and In were applied as internal standards at final concentrations of 20 and 4 μ/L, respectively. The iodine content in the serum diluted at a ratio of 1:51.5 with TMAH solution was directly analyzed by inductively coupled plasma mass spectrometry (ICP-MS), iCAP Q with CASX260 (Thermo Fisher Scientific, Waltham, MA, USA). For analytical quality control, three biological reference materials (matrix: serum), that is, QM-S-Q 1825 (I:54.4 μg/L), QM-S-Q 2007 (I:93.6 μg/L), and QM-S-Q 2008 (I:135 μg/L) (Center de toxicologie/Institut National de Sante Publique Quebec (INSPQ), Quebec, Canada) were disintegrated in the samples and measured after every 20th sample. The sensitivity of this assay was 3 μg/L. The intra- and inter-assay coefficients of variation were 2.0–2.6% and 1.8–6.3%, respectively. All analytical procedures were performed at the Shiohama factory, Daiichi Yakuhin Sangyo Co., Ltd., and at the Central Research Laboratory, Tsukuba, Kotobiken Medical Laboratories, Inc.

### Leukocyte isolation and B cell culture

The draining lymph nodes (cervical and submandibular lymph nodes) and spleen were crushed on a cell strainer. Red blood cells in the spleen were removed using RBC lysis buffer (BioLegend) according to the manufacturer’s instructions. For B cell culture, splenic B cells were sorted using an iMag cell separation system (BD) and biotin-conjugated antibodies (anti-CD3ε, anti-CD11b, anti-Gr1, anti-NK1.1, and anti-TER119). To induce the IgE class-switch of B cells, 250 ng/mL anti-CD40 antibody (clone: 1C10, Invitrogen, Thermo Fisher Scientific) and 25 ng/mL L-4 (BioLegend) were added to 2 × 10^5^ B cells for 3 days. Necrosulfonamide (1 μM; Cayman Chemical Company, Ann Harbor, MA, USA), Z-YVA-FMK (1 μM; BioVision Inc., Waltham, MA, USA), and ferrostatin-1 (1 μM; Sigma-Aldrich) were administered at the onset of culture.

### Flow cytometric analysis

The separated leukocytes were stained with fluorescent-conjugated antibodies purchased from BioLegend, BD Biosciences (Franklin Lakes, NJ, USA), Thermo Fisher Scientific, and Tonbo Biomedicine as follows: anti-CD3, anti-CD4, anti-CD8α, anti-CD11b, anti-CD11c, anti-CD45, anti-CD45R(B220), anti-CD95, anti-CD117(c-kit), anti-CXCR5, anti-FcεRI, anti-GL-7, anti-IgE, anti-Ly6C, anti-Ly6G, anti-PD-1, and anti-Siglec-F (Supplementary Table 1). Dead cells were stained with SYTOX blue or 7-AAD (BioLegend). Apoptotic cells were stained using an annexin V apoptosis detection kit (Biolegend) following the manufacturer’s protocol. To detect intracellular ROS and ferrous iron, ROS Assay Kit-High Sensitive DCFH-DA (DOJINDO) and FerroFarRed (Goryo Chemical, Inc.), were used, respectively, following the manufacturer’s protocol. The cells were analyzed using LSRII (BD Biosciences).

### Quantitative polymerase chain reaction

Total mRNA was extracted and reverse-transcribed using TRIzol LS Reagent (Invitrogen) and ReverTra Ace (Toyobo, Osaka, Japan), respectively. Gene expression was measured using the SsoAdvanced Universal SYBR Green Supermix (Bio-Rad Laboratories, Hercules, CA, USA) and CFX Connect (Bio-Rad). Primer sequences were described in Supplementary Table 2.

### Statistical analyses

Statistical comparisons between the two groups were performed using the Student’s *t*-test, Welch’s *t*-test, or Mann–Whitney’s *U*-test. When the values are not a normal distribution by Shapiro–Wilk test, the Mann–Whitney *U*-test was used for the analysis. When the values show the normal distribution and the heteroscedasticity by F-test, Welch’s *t*-test was used. If the values showed normal distribution and homoscedasticity, Student’s *t*-test was employed for the analysis. Statistical comparisons between more than three groups were evaluated by one-way (single factor) or two-way (dual factor) analysis of variance (ANOVA) test with Tukey’s multiple comparisons test. The correlation was analyzed using linear regression analysis and Pearson’s correlation analysis. Statistical analyses were performed using GraphPad Prism or R. Results were considered statistically significant when P < 0.05.

### Ethics declarations

All animal experiments were approved by the Animal Research Committee of Keio University and were performed in accordance with relevant guidelines and regulations (Institutional Guidelines on Animal Experimentation at Keio University). This study is reported in accordance with ARRIVE guideline.

## Supplementary Information

Supplementary Information.

## Data Availability

The data in this study are available from the corresponding author K.H., upon reasonable request.
