# Supplementary material for: Dietary iodine attenuates allergic rhinitis by inducing ferroptosis in activated B cells
Source: Sci Rep. 2023 Apr 3;13:5398. doi: 10.1038/s41598-023-32552-1 (PMC10070403; doi:10.1038/s41598-023-32552-1)
Supplement: Supplementary file 1 — Supplementary Information. [file 41598_2023_32552_MOESM1_ESM.pdf]

Supplementary Figure 1

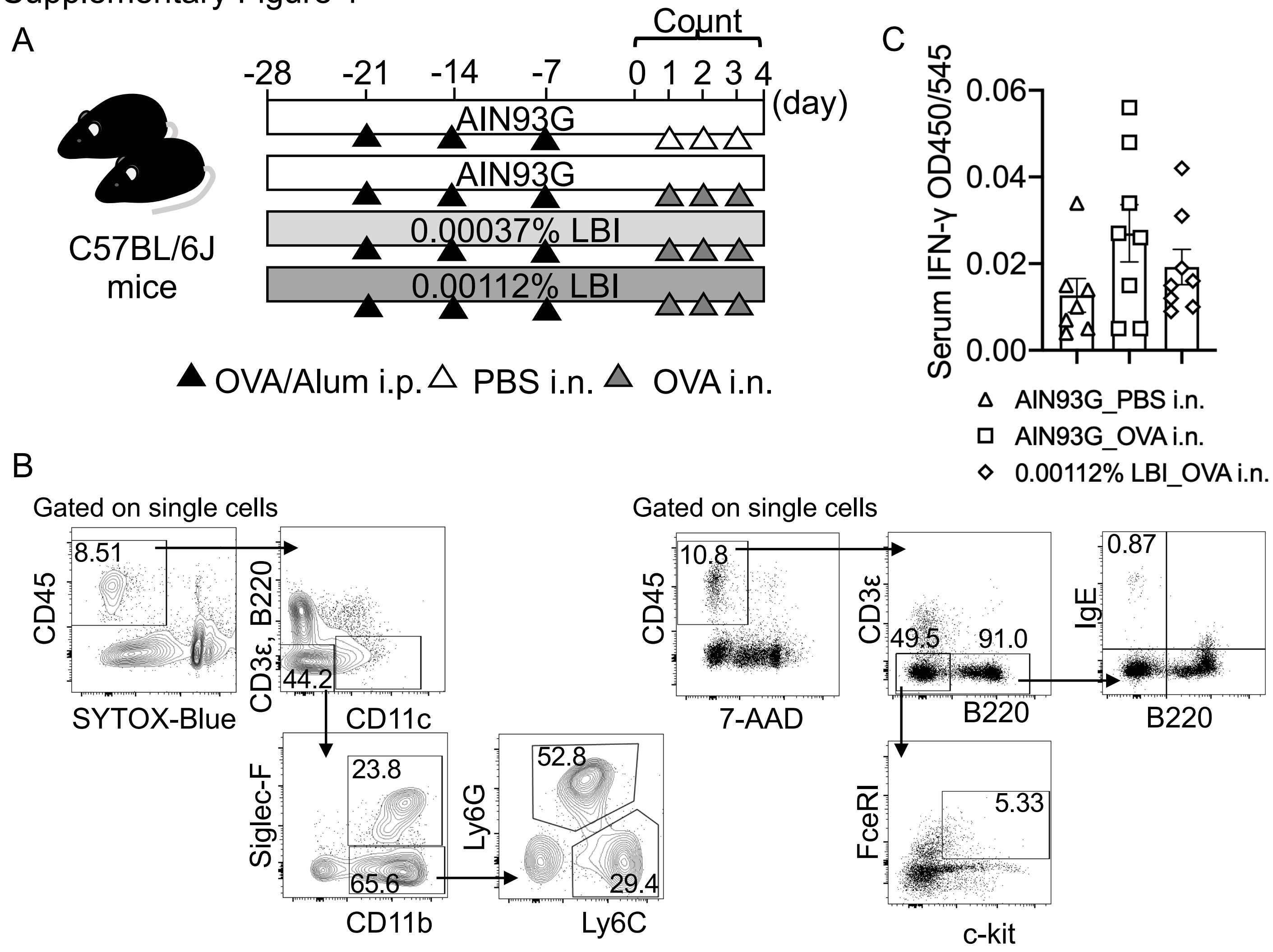

(A) A scheme of the experimental protocol of allergic arthritis model. Male C57BL6J mice were intraperitoneally sensitized by OVA/Alum for totally 3 times before assessment of nasal symptoms. Mice were intranasally exposed by PBS or OVA/PBS at day 1, 2, and 3, and sneezing and nasal rubbing were counted for 5 min after the exposures. (B) FCM strategy in allergic rhinitis model. (C) Serum IFN-γ levels at the last experimental day were measured. The data were pooled from two different experiments and shown as the means ± SEM. (n = 7, 8, 8) One-way ANOVA test with Tukey's multiple comparisons test. n.s.: not significant; $*p < 0.05$, $**p < 0.01$

Supplementary Figure 2

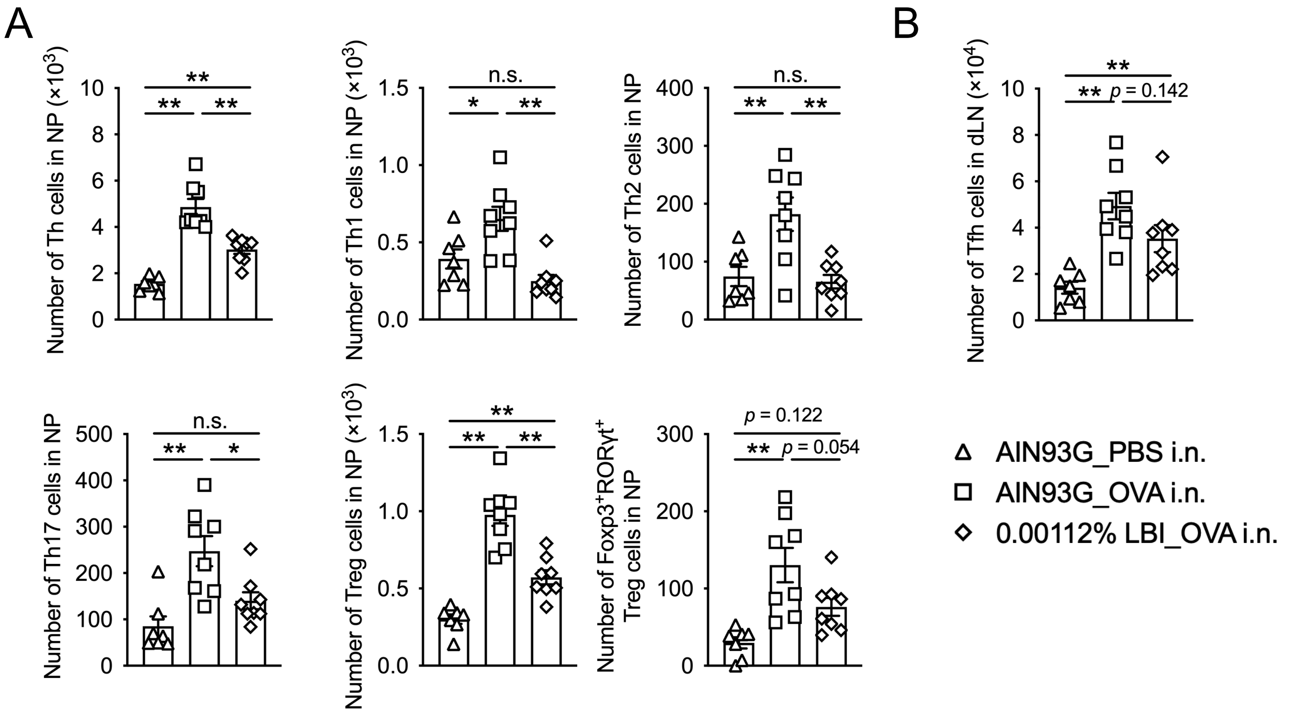

(A) The number of Th cells, T-bet^{+}Th1 cells, Gata3^{+}Th2 cells, ROR-γt^{+}Th17 cells, Foxp3^{+}Treg cells, and ROR-γt^{+}Foxp3^{+}Treg cells were measured in the nasal passage (NP) of allergic mice. (B) The number of Tfh cells in draining lymph node (dLN) in allergic mice. The data were pooled from two different experiments and are shown as the means ± SEM (n = 7, 8, 8, 8). One-way ANOVA with Tukey's multiple comparison test. n.s.: not significant, *$p < 0.05$, **$p < 0.01$.

Supplementary Figure 3

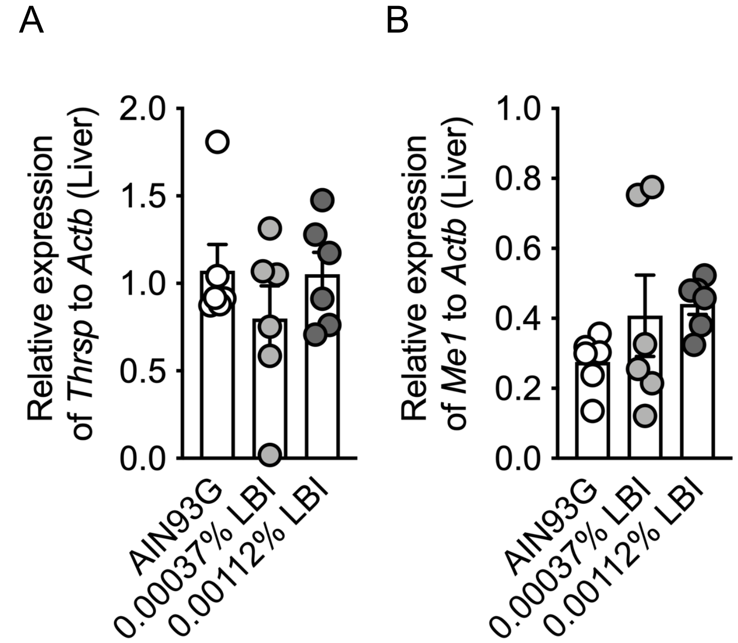

(A, B) Adult male C57BL/6J mice were fed each diets for 3 weeks. The expression levels of T3-responsive genes, *Thrsp* (A) and *Me1* (B), were measured. Data are means ± SEM from two independent experiments (n = 6, 6, 6). One-way ANOVA with Tukey multiple comparison test. *$p < 0.05$, n.s., not significant.

Supplementary Figure 4

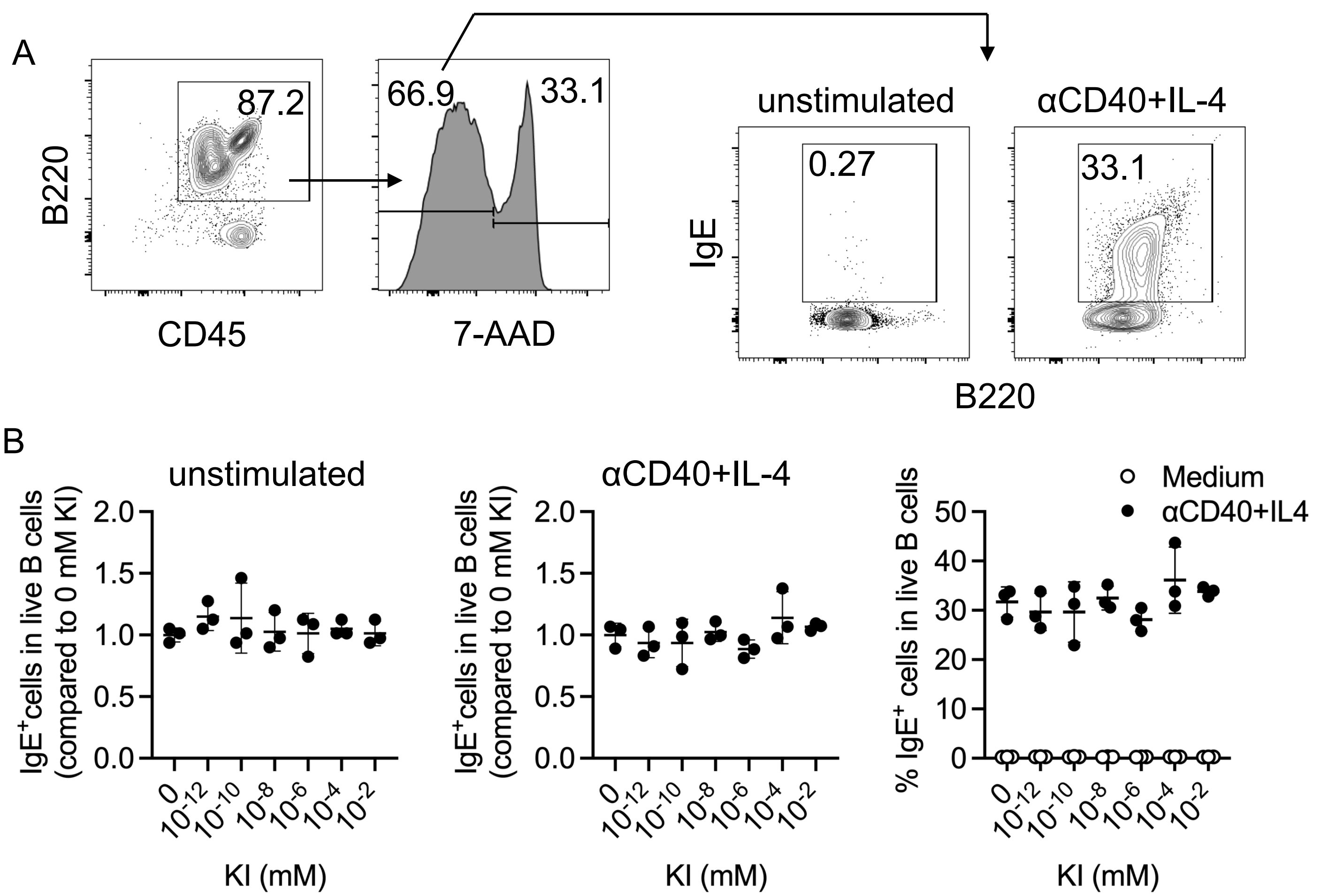

(A) FCM plot for analyzing IgE class-switching. The first plot is gated on the single cell population. (B) Fold increases of the ratio of IgE^{+}cells in live B cells cultured with KI under unstimulated (left) or stimulated (middle) conditions. The right panel shows the frequency of IgE^{+} cells in $CD45^{+}B220^{+}$ B cells. Similar results were obtained from two-independent experiments. One-way ANOVA with Tukey multiple comparison test (right and middle panels).

Supplementary Figure 5

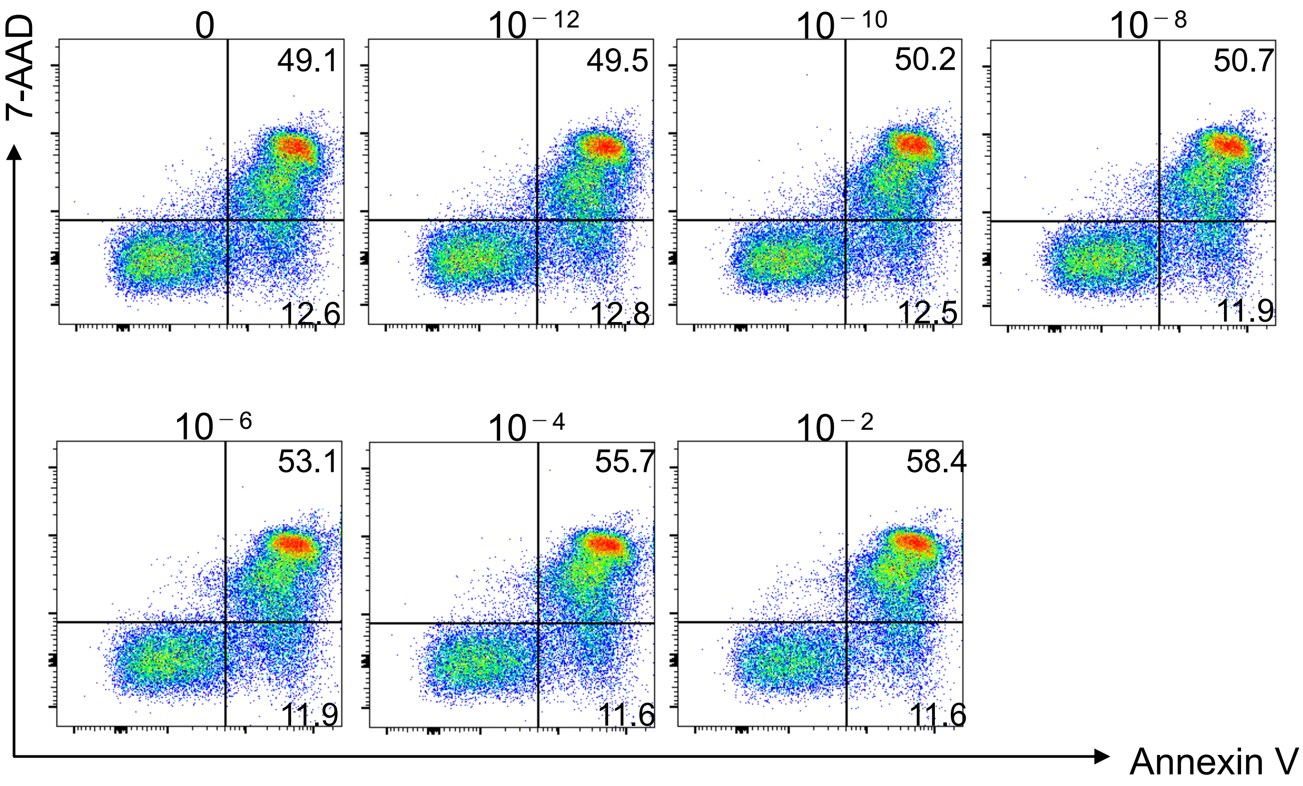

FCM plot for analyzing apoptosis. The plots were gated on $B220^{+}CD45^{+}$single cell populations. The results are interpreted as follows: Annexin V^{+}7-AAD^{-}cells; early apoptotic cells, Annexin V^{+}7-AAD^{+}cells; late apoptotic cells.

Supplementary Figure 6

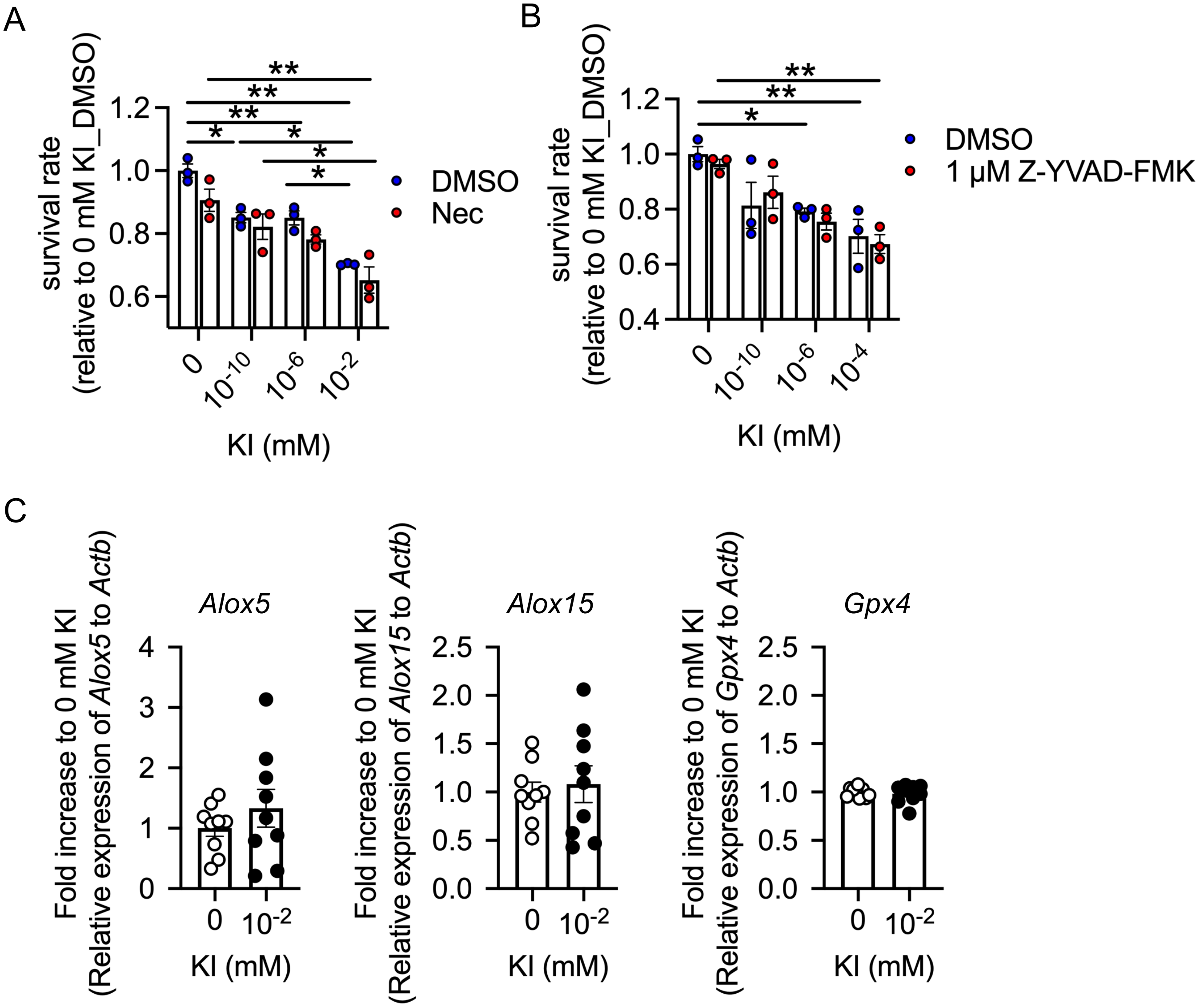

(A, B) Splenic B cells were cultured with 1 μM necrosulfonamide (Nec: A) or 1 μM Z-YVAD-FMK. Data (n = 3) were calculated by the Two-way ANOVA with Tukey multiple comparison test. Similar results were obtained from two independent experiments. (C) The expression of Alox5, Alox15, and Gpx4 in anti-CD40-stimulated B cells was detected. Data (n = 9) were pooled from three independent experiments and calculated by Student' t-test (*Alox15* and *Gpx4*) and Welch's t-test (*Alox5*).

Supplementary Figure 7

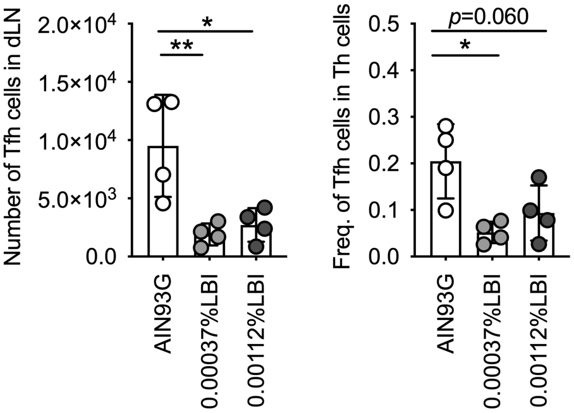

The number and frequency of Tfh cells in dLN of LBI diet-fed mice for 3 weeks. Representative data from two independent experiments are shown as the means ± SD (n = 4 for each group) One-way ANOVA with Tukey's multiple comparison test. n.s.: not significant, *$p < 0.05$, **$p < 0.01$.

Supplementary Figure 8

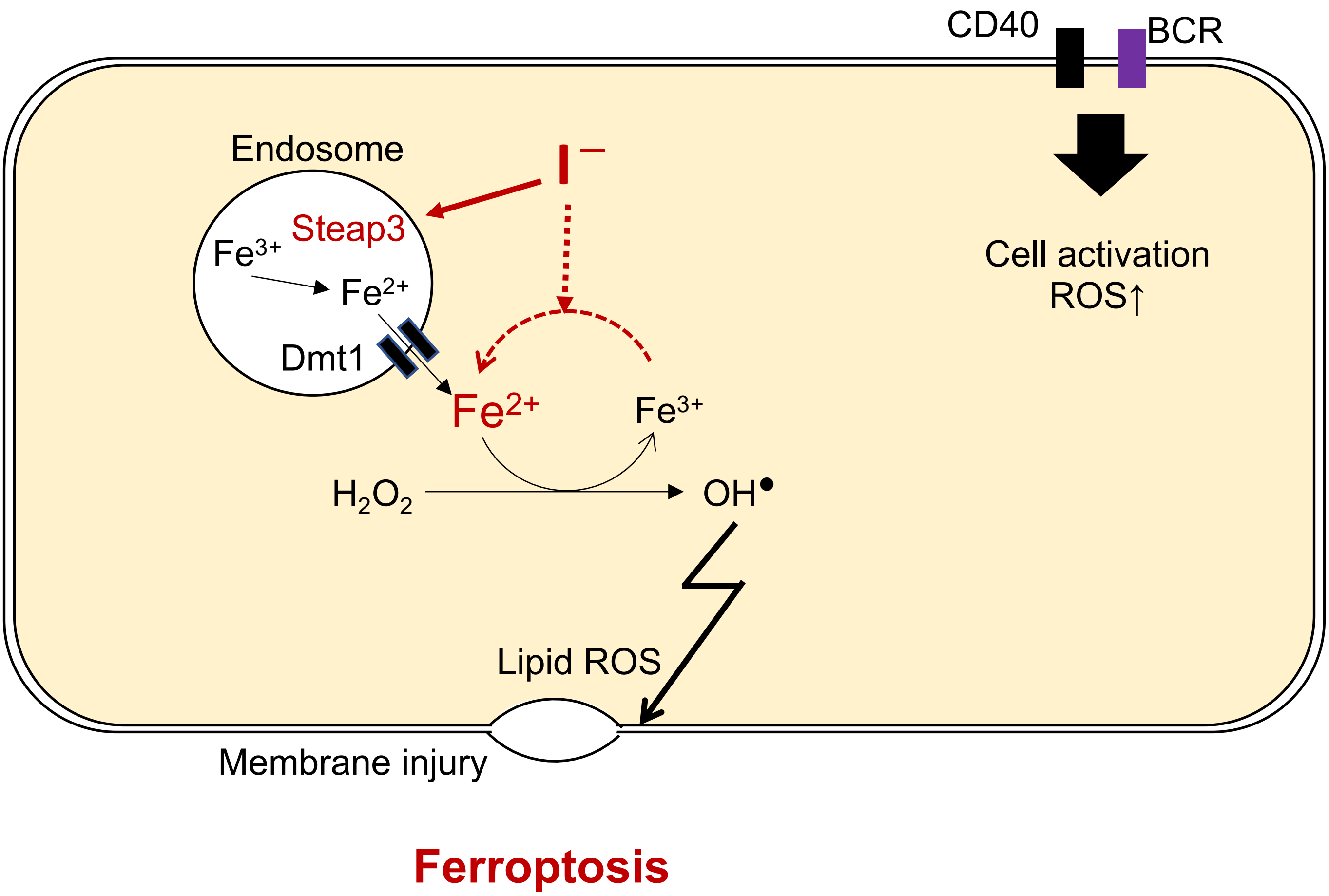

A scheme of this study. Iodide increases ferrous iron (Fe^{2+}) by elevating the expression level of Steap3 in the endosome. Increased Fe^{2+} accelerates hydroxyl radical production via the Fenton reaction, and eventually induces accumulation of lipid ROS. In addition, iodide chemically converts ferric iron (Fe^{3+}) to Fe^{2+}. Given that BCR-induced cell activation also increased intracellular ROS, iodide may give activated B cells a push to the ferroptosis.

Supplementary Table 1. Information on the antibodies used this study

antibody	conjugated	Clone	Campany	Cat#
anti-CD3ε	biotin	145-2C11	eBioscience	13-0031-85
anti-CD11b	biotin	M1/70	BioLegend	101204
anti-Gr1	biotin	RB6-8C5	BioLegend	108404
anti-NK1.1	biotin	PK136	TONBO	30-5941-U500
anti-TER119	biotin	TER-119	BioLegend	116204
anti-CD3ε	FITC	17A2	TONBO	35-0032-U500
anti-CD3ε	PE-Cy7	145-2C11	invitrogen	1993626
anti-CD4	APC-H7	GK1.5	BD Biosciences	560181
anti-CD8α	PE	53-6.7	BioLegend	100708
anti-CD11b	PerCP-Cy5.5	M1/70	invitrogen	2005223
anti-CD11c	PE-Cy7	HL3	BD Biosciences	558079
anti-CD45	BV510	30-F11	BioLegend	103138
anti-CD45	redFluor710	30-F11	TONBO	80-0459
anti-CD45R	FITC	RA3-6B2	eBioscience	11-0452-85
anti-CD45R	APC-Cy7	RA3-6B2	invitrogen	2272766
anti-CD95	APC-R700	Jo2	BD Biosciences	565130
anti-CD117	BV421	2B8	BioLegend	105827
anti-FcεRIα	FITC	Mar-1	BioLegend	134305
anti-GL-7	Alexa Flour647	GL7	BioLegend	144606
anti-Ly6C	APC	AL-21	BD Biosciences	560595
anti-Ly6G	APC-Cy7	1A8	BioLegend	127624
anti-IgE	PE	RME-1	BioLegend	406908
anti-Siglec-F	PE-CF594	E50-2440	BD Biosciences	562757

Supplementary Table 2. Primer sequences used for qPCR

gene name	Sequence	ForR
Alox5	5’-ACACTGAAGACACCCCACGG-3’	Forward
Alox5	5’-ACGTCTGTGCTGCTTGAGGA-3’	Reverse
Alox15	5’-CTTCACAGGTTCTGGGGACAA-3’	Forward
Alox15	5’-AGGCTTTTCCAGCAGGTCAC-3’	Reverse
Gpx4	5’-GCCGTCTGAGCCGCTTACTT-3’	Forward
Gpx4	5’-GATGCACACGAAACCCCTGT-3’	Reverse
Fth1	5’-CCGAGATGATGTGGCTCTGAA-3’	Forward
Fth1	5’-CAGTCATCACGGTCTGGTTTCTTT-3’	Reverse
Ftl1	5’-ACTTAGAGCAGCGCCTTGGA-3’	Forward
Ftl1	5’-GTAGGAGCTAACCGCGAAGA-3’	Reverse
Actb	5’-GATCTGGCACCACACCTTCT-3’	Forward
Actb	5’-GGGGTGTTGAAGGTCTCAAA-3’	Reverse
Steap3	5'-GAGGTCATCTTTGTGGCCGT-3'	Forward
Steap3	5'-TCCGTGGGGTTGCTTACATC-3'	Reverse
Slc11a2	5'-GTGGCGGAGCCGAATCCTAT-3'	Forward
Slc11a2	5'-CGCCATCGTCTGGCATCTTTTC-3'	Reverse
